# Role of atherogenic indices in predicting infertility in polycystic ovary syndrome

**DOI:** 10.1590/1806-9282.20241460

**Published:** 2025-08-08

**Authors:** Beyzanur Kahyaoğlu, Esra Keles, İsmail Bağlar, Mehmet Güçlü, Buket Yiğit Çivi, Sahra Sultan Kara, Gizem Elif Dizdaroğulları

**Affiliations:** 1 Department of Obstetrics and Gynecology, Kartal Dr. Lütfi Kırdar City Hospital, University of Health Sciences – İstanbul, Turkey.; 2 Department of Gynecologic Oncology, Kartal Dr. Lütfi Kırdar City Hospital, University of Health Sciences – İstanbul, Turkey.; 3 Department of Maternal Fetal Medicine, Kartal Dr. Lütfi Kırdar City Hospital, University of Health Sciences – İstanbul, Turkey.

**Keywords:** Polycystic ovary syndrome, Infertility, Insulin resistance, Reproductive health, Metabolic syndrome, Triglyceride, Lipid metabolism

## Abstract

**OBJECTIVE:**

The aim of the study was to assess the predictive value of the triglyceride-glucose index and atherogenic indices for infertility in women with polycystic ovary syndrome.

**METHODS:**

This prospective, single-center, non-randomized observational study was conducted on 279 women diagnosed with polycystic ovary syndrome from May to December 2023. Women with polycystic ovary syndrome were grouped into two groups: those with infertility and those without infertility. Demographic, hormonal, and clinical parameters were studied. The statistical study employed IBM SPSS (Statistical Package for the Social Sciences) Statistics 22 to analyze the distribution of variables, assessing normality and comparing categorical and continuous data. Descriptive statistics were computed, with categorical and continuous data compared using appropriate tests (chi-square, Student's t-test, and Mann-Whitney U). Multivariable logistic regression identified independent predictors of infertility, with a significance level set at 0.05.

**RESULTS:**

Infertile women with polycystic ovary syndrome had significantly higher plasma levels of dehydroepiandrosterone sulfate (p=0.001), testosterone (p=0.005), insulin (p=0.041), Homeostasis Model Assessment of Insulin Resistance (p=0.029), prolactin (p=0.018), triglycerides (p<0.001), triglyceride/high-density lipoprotein (p=0.001), atherogenic index of plasma (p=0.011), triglyceride-glucose index (p=0.001), and lipoprotein combine index (p=0.007) compared to the fertile women with polycystic ovary syndrome. Correlation analysis showed that the triglyceride-glucose index correlated with the Homeostasis Model Assessment of Insulin Resistance (r=0.402, p<0.001) and total testosterone (r=0.191, p=0.001). Multivariable analysis identified age (OR 1.189, 95%CI 1.122–1.263, p<0.001), prolactin (OR 1.040, 95%CI 1.004–1.077, p=0.029), and triglyceride-glucose index (OR 2.473, 95%CI 1.404–4.177, p<0.001) as independent predictors of infertility.

**CONCLUSION:**

This study suggests a more atherogenic lipid profile in infertile women with polycystic ovary syndrome, suggesting a significant link between dyslipidemia and infertility. The triglyceride-glucose index proves to be a reliable, non-invasive marker of insulin resistance and may aid in identifying women at higher risk for infertility, facilitating earlier, targeted interventions.

## INTRODUCTION

Infertility is increasingly being recognized as a social issue and civilization disease of the 21st century^1^. The extent of this problem is difficult to estimate, but it is known that 8–12% and even up to 18% of couples of reproductive age worldwide suffer from infertility^2^.

Polycystic ovary syndrome (PCOS) represents the most common etiology of female infertility. The underlying mechanisms of this condition remain incompletely elucidated. Nevertheless, it is broadly acknowledged that a complex interplay of immunometabolic factors significantly contributes to its pathogenesis, ultimately resulting in infertility^3,4^. Furthermore, PCOS is associated with an elevated risk of metabolic dysfunction, insulin resistance (IR), insufficient progesterone secretion, and oxidative stress, which may lead to various health complications, including coronary heart disease, cancer, autoimmune disorders, dyslipidemia, and infertility^5^.

Recent studies have indicated that elevated serum lipid concentrations may be linked to infertility in women with PCOS^6^. Dyslipidemia, frequently observed in this population, has been associated with impaired ovarian function and decreased fertility^7^. Serum lipid levels correlate with clinical pregnancy rates, live birth outcomes, and miscarriage rates among women with PCOS^8^. Understanding the role of atherogenic indices in predicting infertility could have important clinical implications. By identifying women at increased risk of infertility based on their lipid profiles, healthcare providers may be able to implement more targeted interventions to enhance reproductive outcomes. Additionally, addressing dyslipidemia and its effects on fertility may offer new strategies for managing PCOS-related infertility.

To date, many studies have used metabolic lipid or atherogenic indices to analyze cardiometabolic risk in women with PCOS. However, the relationship between the triglyceride-glucose index (TyG), atherogenic indices, and infertility in this population has not been investigated. Therefore, this study aims to examine the potential utility of novel atherogenic indices, along with the TyG, in evaluating infertility among women diagnosed with PCOS.

## METHODS

The prospective, single-center, non-randomized observational study was conducted on 279 women with PCOS who sought treatment at the obstetrics and gynecology department of a tertiary care hospital between May and December 2023. The diagnostic criteria for PCOS were established in accordance with the Rotterdam criteria^9^.

The participants were categorized into two groups: those experiencing infertility and those without infertility. The etiologies of infertility were determined through a comprehensive approach that included clinical assessments, laboratory tests, hysterosalpingography, and hysteroscopic or laparoscopic procedures.

The research study received approval from the Research Ethics Committee under the approval number 2023/514/250/16 and complied with the ethical guidelines outlined in the Declaration of Helsinki. Before enrollment in the study, all participants provided written informed consent.

The exclusion criteria for the study included the following: lack of patient consent; pregnancy; use of hormonal contraceptives, glucocorticosteroids, or antihyperlipidemic agents, as well as medications that influence glucose metabolic pathways; a prior diagnosis and ongoing treatment for diabetes or thyroid disease; a history of depression; and other potential causes of hyperandrogenism or ovulatory dysfunction.

Fasting venous blood samples were obtained on the third day of the menstrual cycle between 8:00 and 10:00 in the morning. Plasma glucose and serum lipid levels were determined using the spectrophotometric method with an autoanalyzer assay (Beckman Coulter AU 5800, MN, USA). Serum insulin was measured using direct chemiluminescence (Siemens, New York, USA). Furthermore, the serum hormonal parameters were quantified using an automated chemiluminescence immunoassay system, the UniCel DxI 800 autoanalyzer (Beckman Coulter^®^, CA, USA).

Calculated parameters related to insulin sensitivity are as follows:


$${\text{Body mass index}}^{10};=\frac{weight\left(kg\right)}{\left[height\left(m\right)\right]2}$$

Homeostasis Model Assessment of Insulin Resistance


$$\begin{array}{c} {\left(\text{HOMA}-\text{IR}\right)}^{11};=\frac{Fasting\;glucose(\frac{mmol}{L})\times Fasting\;insulin\left(mIU/mL\right)}{22.5}\\ TyG\;index^{11}];=Ln[\frac{fasting\;triglycerides\left(\frac{mg}{dL}\right)\times fasting\;glucose\left(\frac{mg}{dL}\right)}{2} \end{array}$$

TyG-BMI^12^: TyG index×Body mass index;


$$\begin{array}{l} \text{Castelli}\prime {\text{s risk index-I}}^{13};=\frac{Total\;cholesterol\left(\frac{mg}{dL}\right)}{High-density\;Lipoprotein\;Cholesterol\left(\frac{mg}{dL}\right)}\\ \text{Castelli}\prime {\text{s risk index-II}}^{13};=\frac{Low-density\;Lipoprotein\;Cholesterol\left(mg/dL\right)}{High-density\;Lipoprotein\;Cholesterol\left(mg/dL\right)}\\ {\text{TG/HDL-C}}^{13};=\frac{Triglycerides\left(mg/dL\right)}{High-density\;Lipoprotein\;Cholesterol\left(mg/dL\right)} \end{array}$$

Atherogenic index of plasma


$${\left(\text{AIP}\right)}^{13}=\log[\frac{Triglycerides\left(mg/dL\right)}{High-density\;Lipoprotein\;Cholesterol\left(mg/dL\right)}]);$$

Atherogenic coefficient^13^=(Serum total cholesterol (mg/dL)−Serum HDL-C (mg/dL)/HDL-C (mg/dL).

Lipoprotein combine index


$${\left(\text{LCI}\right)}^{14}=\left(\times \right)\frac{Total\;Cholesterol\left(\frac{mmol}{L}\right)\times Triglycerides\left(\frac{mmol}{L}\right)\times Low-density\;Lipoprotein\;Cholesterol\left(mmol/L\right)}{High-density\;Lipoprotein\;Cholesterol\left(mmol/L\right)}$$

### Statistical analysis

The IBM SPSS (Statistical Package for the Social Sciences) Statistics 22 software was employed to conduct statistical analyses. The normality of the variable distributions was evaluated using the Kolmogorov-Smirnov test. Descriptive statistics are presented as the mean±standard deviation (SD) for continuous variables and as counts and percentages for categorical data. The chi-square test was utilized to compare categorical variables, while the Student's t-test and Mann-Whitney U test were applied for parametric and non-parametric continuous variables, respectively. Correlations among continuous variables were assessed using both Spearman's and Pearson's correlation coefficients, depending on the normality of the data distribution. Multivariable logistic regression analysis was conducted to identify independent predictors of infertility. The analysis was performed using the enter method, where all variables that demonstrated statistical significance (p≤0.05) in the univariate analysis were included in the model. A significance level of 0.05 was established.

## RESULTS

The study included 279 patients diagnosed with PCOS. These patients were divided into two groups: those who remained non-pregnant after 1 year of unprotected intercourse (n=129) and those who achieved pregnancy without any medical intervention (n=145). Table 1 presents a summary of the baseline demographics, clinical characteristics, and laboratory findings of the participants. The mean age of infertile women with PCOS was found to be significantly higher than that of fertile women with PCOS (p<0.001). Furthermore, the PCOS patients experiencing infertility exhibited significantly elevated plasma levels of dehydroepiandrosterone sulfate (DHEAS) (p=0.001), testosterone (p=0.005), insulin (p=0.041), HOMA-IR (p=0.029), prolactin (p=0.018), triglycerides (p<0.001), triglyceride to high-density lipoprotein (TG/HDL) ratio (p=0.001), AIP (p=0.011), TyG index (p=0.001), and LCI levels (p=0.007) when compared to fertile women with PCOS.

**Table 1:** Comparison of clinical and laboratory characteristics between fertile and infertile polycystic ovary syndrome patients.

Variables	Fertile PCOS (n=145)	Infertile PCOS (n=129)	p-value
Age (years)	28.1±4.9	32.5±5.1	**<0.001** *
BMI (kg/m^2^)	28.1±0.6	27.5±0.5	0.392*
Total testosterone (ng/dL)	33 [22–39]	37 [25–50]	0.468*
DHEAS (μg/dL)	208.3±84.7	244.4±95.2	0.441**
Insulin (m IU/mL)	11.3±4.1	13.6±6.3	0.06**
HOMA-IR	2.5±1.4	3.0±2.3	**0.005** **
Cholesterol (mg/dL)	171.4±35.7	178.8±31.3	**0.001** *
LDL-C (mg/dL)	99.8±36.2	105.6±26.1	0.181**
HDL-C (mg/dL)	50.5±11.3	52.7±12.2	**0.041** *
Triglyceride (mg/dL)	67 [50–103]	85 [64–133]	**0.029** *
TG/HDL-C	1.3 [0.9–2.2]	1.7 [1.1–2.8]	0.075*
Castelli Risk Index-I	3.5±1.1	3.5±0.9	0.140*
Castelli Risk Index-II	2.1±0.9	2.1±0.7	0.112*
AIP	0.13 [-0.14 to 0.34]	0.23 [0.05–0.45]	**<0.001** **
Atherogenic coefficient	2.5±1.1	2.5±0.9	**0.011** **
Fasting glucose	89.4±17.7	89.3±12.8	0.962*
TyG index	8.0±0.5	8.3±0.6	0.629*
TyG-BMI	228.8±61.6	229.2±62.1	**0.011** **
LCI	24.601 [13.387–40.101]	29.332 [192.250–62.091]	0.962*
PRL (μg/L)	13.4 [10.3–18.1]	15.8 [11.2–21.5]	0.986*

* Student's t-test.

** Mann-Whitney U test.

BMI: body mass index; E2: estrogen; HDL-C: high-density lipoprotein cholesterol; HOMA-IR: homeostasis model assessment for insulin resistance; LDL-C: low-density lipoprotein cholesterol; PCOS: polycystic ovary syndrome; TG: triglycerides; TyG: triglyceride glucose; TyG-BMI: triglyceride glucose body mass index; PRL: prolactin; DHEAS: dehydroepiandrosterone sulfate. LCI: lipoprotein combine index; AIP: atherogenic index of plasma. Data are expressed as median (Q1–Q3), mean±SD, or number (percentage) where appropriate. A p-value of <0.05 indicates a significant difference. Statistically significant p-values are in bold. Data are expressed as median (Q1–Q3), mean±SD, or number (percentage) where appropriate. A p-value of <0.05 indicates a significant difference. Statistically significant p-values are in bold.

Correlation analysis revealed a moderate positive correlation between the TyG index and HOMA-IR (r=0.402, p<0.001) and a weak positive correlation between the TyG index and total testosterone (r=0.191, p<0.001). The correlation coefficients in Pearson's and Spearman's correlation analyses are presented in Table 2. The significant variables in the t-test/Mann-Whitney U test, including age, total testosterone, DHEAS, HOMA-IR, prolactin, and TyG index, were evaluated with multivariable binary logistic regression analysis.

**Table 2:** Correlation between triglyceride-glucose and different variables.

Variables	R	p-value
Age	0.056*	0.357
Total testosterone	0.191**	0.001
DHEAS	0.023*	0.701
HOMA-IR	0.402*	<0.001
PRL	-0.064**	0.290
AIP	0.908**	<0.001
TG/HDL	0.908**	<0.001
LCI	0.762**	<0.001

* Pearson correlation coefficient.

** Spearman correlation coefficient.

DHEAS: dehydroepiandrosterone sulfate; HOMA-IR: homeostasis model assessment for insulin resistance; PRL: prolactin; LCI: lipoprotein combine index; AIP: atherogenic index of plasma; PCOS: polycystic ovary syndrome; TG: triglycerides; HDL: high-density lipoprotein. A p-value of <0.05 indicates a significant difference.

Although the AIP, TG/HDL, and LCI were found to be significant in the univariate analysis, they were excluded from the multivariate analysis due to a strong correlation with the TyG index. The multivariate logistic regression analysis indicated that age (OR 1.189, 95%CI 1.122–1.263, p<0.001), prolactin (OR 1.040, 95%CI 1.004–1.077, p=0.029), and the TyG index (OR 2.473, 95%CI 1.404–4.177, p<0.001) were identified as independent predictive factors for infertility in patients diagnosed with PCOS (Table 3).

**Table 3:** Multivariate logistic regression analysis for infertile patients with polycystic ovary syndrome.

Variables	OR	95%CI	p-value
Age	1.189	1.122–1.263	<0.001
Total testosterone	0.999	0.990–1.010	0.815
DHEAS	1.004	0.998–1.010	0.109
HOMA-IR	0.973	0.825–1.153	0.744
PRL	1.040	1.004–1.077	0.029
TyG index	2.423	1.404–4.177	0.001

DHEAS: dehydroepiandrosterone sulfate; HOMA-IR: homeostasis model assessment for insulin resistance; PRL: prolactin; CI: confidence interval; OR: odds ratio; TyG: triglyceride glucose. A p-value of <0.05 indicates a significant difference.

## DISCUSSION

The findings of this study suggest that the TyG index may be a promising marker of insulin sensitivity, as evidenced by its significant correlation with HOMA-IR. Factors such as older age, elevated DHEAS levels, higher insulin, HOMA-IR, triglycerides, and prolactin levels were notably different between the two groups, all of which contribute to infertility in women with PCOS. Additionally, elevated levels of DHEAS, testosterone, insulin, prolactin, triglycerides, TG/HDL, AIP, and LCI in the infertile cohort underscore the multifactorial nature of infertility in PCOS. To the best of our knowledge, this investigation represents the first in the literature to explore the association between atherogenic indices and fertility in women with PCOS.

Despite its widespread application, the limitations of the HOMA-IR can be attributed to the inherent complexities associated with insulin measurement^15,16^. In this context, the TyG index has emerged as a promising alternative surrogate marker for IR. Research conducted by Bonora et al.^16^ and Vasques et al.^17^ demonstrated comparable correlations between the TyG index and established methodologies, such as the euglycemic-glucose clamp and hyperglycemic clamp, thereby indicating its potential as a readily accessible and reliable alternative. These findings are consistent with prior research and further support the TyG index as a valid indicator of insulin sensitivity, positioning it as a potentially valuable non-invasive tool for clinical assessment.

Numerous studies have established the utility of the TyG index in identifying and predicting various metabolic and hormonal abnormalities associated with PCOS^18^. Nevertheless, there is a knowledge gap in the existing literature concerning the application of the TyG index for fertility assessment in women diagnosed with PCOS. Our findings indicate that the TyG index effectively distinguishes between fertile and infertile women with PCOS. Furthermore, Li et al.^19^ reported a significant correlation between the TyG index adjusted for body mass index (TyG-BMI) and in vitro fertilization outcomes in this population, suggesting that TyG-BMI may serve as a valuable marker for infertility related to PCOS. By facilitating the identification of women with PCOS who are at an increased risk of infertility, the TyG index may enable earlier interventions and potentially enhance reproductive outcomes.

Women diagnosed with PCOS frequently present with a dyslipidemic profile characterized by elevated triglycerides (TG) levels and reduced HDL levels, both of which are recognized risk factors for cardiovascular disease^3,5,20^. This dyslipidemia has been associated with an increased risk of infertility among women with PCOS^21^. Our research corroborates these findings, revealing elevated levels of TG, the TG/HDL ratio, AIP, and LCI in infertile women with PCOS. However, contrary to expectations, our findings indicated higher HDL levels in infertile women with PCOS. This may be attributed to the small sample size. These findings also suggest a potential link between atherogenic dyslipidemia and infertility in PCOS. One of the possible explanations might be that the elevated levels of TG, free fatty acids, and oxidized low-density lipoproteins are linked to mitochondrial dysfunction, leading to an increase in reactive oxygen species release. This cascade of events ultimately contributes to ovarian damage and a higher incidence of follicular atresia, thereby impacting fertility^21,22^. Further investigation into the relationship between atherogenic indices and fertility in women with PCOS is crucial for understanding the underlying mechanisms and potential treatment targets. In addition to the lipid profile, exploring the role of inflammation and oxidative stress in PCOS-related infertility could provide valuable insights for the development of personalized therapeutic interventions. Moreover, considering the impact of lifestyle modifications and pharmacological approaches on atherogenic indices and fertility outcomes in women with PCOS may offer new avenues for improving reproductive health in this population.

The results of our study showed elevated levels of DHEAS, testosterone, insulin, and prolactin, highlighting the intricate hormonal imbalances that contribute to infertility in PCOS. This finding suggests that hormonal imbalances, particularly DHEAS, testosterone, and insulin levels, may contribute to the development of infertility in PCOS. One possible explanation for the reproductive failures encountered by women with PCOS is the impact of elevated insulin levels on both ovarian function and endometrial physiology. IR, prevalent in this population, causes hyperinsulinemia, disrupting insulin signaling and glucose uptake in the endometrium, which reduces receptivity to embryo implantation. Moreover, obesity exacerbates IR and induces a pro-inflammatory state, further impairing endometrial function and fertility. Molecular defects in the endometrium, linked to IR and metabolic dysregulation, may contribute to reproductive failures in these patients^23^. Another possible explanation is that the elevated insulin levels in women with PCOS can stimulate the ovaries to produce higher levels of testosterone, which in turn disrupts normal ovulation and contributes to infertility^24^. Additionally, the elevated insulin and insulin-like growth factor-1 levels observed in women with PCOS may contribute to the formation of ovarian cysts. These cysts further disrupt ovulation and exacerbate infertility in PCOS^25^. These findings offer a more comprehensive understanding of the intricate link between hormonal imbalances and infertility in PCOS, shedding light on potential targets for therapeutic interventions that may improve reproductive outcomes in women with PCOS.

### Limitations

A key strength of our study is providing novel insights into the complex interplay between atherogenic indices and infertility in PCOS. One key limitation of our study is the lack of an Oral Glucose Tolerance Test (OGTT) to exclude diabetes mellitus among participants. Incorporating the OGTT in future studies would provide a more comprehensive assessment of glucose metabolism and allow for better identification of individuals with impaired glucose tolerance or early stages of diabetes. Additionally, our study's single-center design and relatively small sample size may limit the generalizability of the findings. Further research is needed to validate our results in larger, multicenter cohorts. Moreover, exploring the underlying mechanisms linking dyslipidemia, IR, and infertility could inform targeted therapeutic strategies aimed at improving reproductive outcomes in this population.

## CONCLUSION

This study reveals a more atherogenic lipid profile in infertile women with PCOS, suggesting a significant connection between dyslipidemia and infertility. The TyG proves to be a reliable, non-invasive marker of IR. Our findings indicate that the TyG index could serve as a valuable tool for identifying women at higher risk of infertility, allowing for earlier and more targeted interventions that may improve reproductive outcomes. Additionally, elevated levels of DHEAS, testosterone, insulin, and prolactin highlight the intricate hormonal imbalances that contribute to infertility in this population. Understanding these relationships could inform the development of tailored therapeutic strategies aimed at enhancing fertility in women with PCOS. Future research should investigate the potential benefits of managing dyslipidemia and IR in improving fertility treatment outcomes.

## Data Availability

The datasets generated and/or analyzed during the current study are available from the corresponding author upon reasonable request.
